# Sowing Date and Seeding Rate Affect Bioactive Compound Contents of Chickpea Grains

**DOI:** 10.3390/ani9080571

**Published:** 2019-08-17

**Authors:** Riccardo Primi, Roberto Ruggeri, Bruno Ronchi, Umberto Bernabucci, Francesco Rossini, Mercedes Martin-Pedrosa, Pier Paolo Danieli

**Affiliations:** 1 Dipartimento di Scienze Agrarie e Forestali, Università della Tuscia, 01100 Viterbo, Italy; 2 Instituto Nacional de Investigacion y Tecnologia Agraria y Alimentaria, Carretera de la Coruna Km. 7.5, 28040 Madrid, Spain

**Keywords:** legume seeds, pulses, soluble carbohydrates, α-galactosides, trypsin inhibitors, inositol phosphates

## Abstract

**Simple Summary:**

Chickpea seeds are commonly used in human diets in many areas of the world, and it can be a valid alternative to soybean meals and energetic feedstuff in animal nutrition. However, its excellent nutritional characteristics are accompanied by the occurrence of some bioactive substances with anti-nutritional effects. Usually, those secondary compounds are inactivated using appropriate processing techniques, but this increases costs and causes a loss of some nutritional traits. It is well-known that grain composition changes according to the genetic type and environmental conditions, and this study suggests that it is possible to modulate the presence of bioactive compounds in the seed also by varying some agronomic parameters, such as the sowing dates and the seeding rate. This study demonstrates that, under Mediterranean climate conditions, spring sowing reduces the content of trypsin inhibitors, and a high seeding rate can depress the content of α-galactosides. A controversial effect of the sowing period was observed on the content of inositol phosphates, probably because of the different climatic conditions during the two-year experimental period. Consequently, agronomic management can modulate the content of anti-nutritional factors, reducing the likelihood that they will avoid feeding treatments.

**Abstract:**

Chickpea grains may represent an alternative to soybean meals and energetic concentrates in animal feeding, as their nutritional value can help to increase the sustainability of livestock systems. Unfortunately, the presence of bioactive compounds with anti-nutritional effects can prevent its direct use, especially in mono-gastrics. It is known that the synthesis of these compounds depends on genetic expression, which is also influenced by growth conditions. The objective of this two-year study was to assess the effect of sowing date (winter versus spring) and seeding rate (70 versus 110 seeds m^−2^) on the accumulation of soluble carbohydrates, α-galactosides, trypsin inhibitors, and inositol phosphates in the grains of two Kabuli cultivars, in the Mediterranean climate. The results showed that seeds collected from winter sowing contained more trypsin inhibitors than those seeded in spring (+ 4%, on average), reaching values between 16.1 and 18.6 TIU mg protein^−1^. The seeding rate affects only the α-galactosides content, which increases (+9%) at lower densities (70 seeds m^−2^). These findings suggest that agronomic management can be used to modulate the content of some anti-nutritional factors in the seeds, even though the genetic characteristics and phenotypic expression, in relation to the climatic conditions, seem to deeply affect the content of all the bioactive compounds investigated.

## 1. Introduction

Chickpea (*Cicer arietinum* L.) is a traditional source of plant proteins for humans, and its use for animal nutrition could provide a range of benefits, both for farmers and feed manufacturers. It could also be particularly interesting for organic agriculture and in mixed farming systems. As other traditional pulses, the chickpea could support the EU to reduce its dependence on imported vegetal proteins, because it can be used as an alternative to soybean [1].

Chickpea is an annual grain legume traditionally cultivated in semiarid tropics (India), Australia, and the Mediterranean regions, and it has recently increased its acreage, extending the cultivation area to higher latitudes [2]. However, low productivity, susceptibility to climate conditions, competition from other remunerative crops, and the emergence of more productive and profit-oriented production systems have seriously reduced the diffusion of this pulse crop in the EU farming systems [3].

Since climate change is expected to bring warmer temperatures and changes in rainfall patterns [4,5], crop adaptation strategies are needed to face this unfavorable scenario, and to reduce the risks on human food security and animal feed supplies. Such adaptation strategies include, shifts in sowing dates [6], breeding for density-neutral cultivars [7], and the identification of alternative or new species tolerant to heat and drought stress [8]. Chickpea is considered one of the most drought-tolerant cool-season pulses [9] and, as other legumes, it has the advantage of improving soil fertility through biological nitrogen fixation, thus helping farmers reduce their use of fertilizers [3]. 

The interesting occurrence of both proteins (up to 29% DM, lysine about 7% of total proteins) and carbohydrates (starch) (up to 59% DM) in suitable proportions, along with fibre, vitamins, and microelements, make this species suitable for nutritional purposes [10,11]. However, as with other leguminous seeds, chickpea contains anti-nutritional compounds (i.e., lectins, phenolic compounds, trypsin inhibitors, inositol phosphates, saponins, oxalic acid, etc.), resulting from the secondary metabolism of the plants, or from flatulence-causing soluble carbohydrates, such as α-galacto-oligosaccharides [12]. These compounds are usually accumulated by plants for chemical defense [13], if ingested in large quantities, could act as anti-nutritional factors (ANFs) and can represent a limiting factor for chickpeas use in animal (and human) nutrition [14]. In fact, especially in monogastrics, nutrient absorption from the gastrointestinal tract can be impaired by the co-presence of ANFs [15], with the onset of detrimental effects on health and growth [16]. On the other hand, these compounds have been found to exhibit antibiotic and health-promoting properties [17]. 

Several studies have shown that raw chickpeas can be used in ruminants, pigs, broilers, laying hens, and fish diets, with different inclusion levels (up to 300 g/kg for ruminant and up 200 g/kg for pigs, birds and fish) [15] with good results. The higher inclusion levels in ruminants are attributable to the fact that the ANFs appear to be inactive in the rumen. Higher inclusion levels of chickpeas in monogastrics diets can be used after removal of the secondary compounds with non-nutritional effect.

To reduce ANFs content of legume seeds, some processing techniques, i.e., soaking, boiling, roasting, microwave cooking, autoclaving, fermentation, micronization, and extrusion could be applied [15,18,19,20], which lead to an unsustainable increase in costs [12] and, sometimes, in the loss of other nutritionals, such as vitamins and minerals [20]. Therefore, it is desirable to develop other strategies to reduce ANFs concentration in chickpea seeds.

Several authors have shown that cultivation conditions affect qualitative traits and yield in various crops and pulses [21,22,23,24], but few studies have been undertaken to investigate the effect on bioactive compounds accumulation in chickpea seeds [25,26].

For example, Nikolopoulou et al. [25] demonstrated that the variable levels of some raffinose family oligosaccharides (RFOs), total tannins and phytic acid in chickpea seeds largely depends on the environmental growing conditions (i.e., area of cultivation and year of cultivation) and variety. 

In a previous work [23], we have shown that the sowing date and seeding rate can influence the yield, proximate composition and total tannins’ content of chickpea seeds. Grain yield, crude protein, crude fat, crude fibre, ash, and total starch were all clearly affected by time (season) of sowing, while the seeding rate had relatively little influence. Winter sowing appeared to be the best choice to maximize yield, while spring sowed plants resulted higher in a crude protein and fibre. 

As the ANFs content of legume grains can be as important as the nutritive principles content, the aim of this two-years study was to evaluate the effects of sowing date and seeding rate on anti-nutritional profile of chickpea seeds.

## 2. Materials and Methods 

Two Italian Kabuli chickpea varieties (Sultano and Pascià) were tested during two growing seasons: 2006/2007 (Y1) and 2007/2008 (Y2). These two chickpea varieties were chosen for their overall resistance to *Ascochyta rabiei* and to represent a range of genetic variations in morphological traits (Sultano: Smooth seed type and erect plant growth habit; Pascià: Rough seed type, semi-erect plant growth habit).

### 2.1. Experimental Set up and Plant Growing Conditions

A randomized complete block design experiment, with three replicates, was performed in both growing seasons (Trial 1) [23]. In addition, two seeding rates, 70 and 110 seeds m^–2^, were tested in Y2 (Trial 2). The experimental fields were established in a Mediterranean area (Central Italy, Tarquinia, 42°13′30′’ N, 11°44′00′’ E, 22 m a.s.l.).

The winter sowing (E1) was carried out on 28th December 2006 (Y1) and 18th December 2007 (Y2). The spring sowing (E2) occurred on 2nd March 2007 (Y1) and 14th March 2008 (Y2). Individual plots (8 m × 1.5 m each) consisted of six rows with a row spacing of 0.3 m and a seeding depth of about 30 mm. Diammonium phosphate (18-46-0) was applied before sowing at rate of 200 kg ha^−1^, and weed control was achieved by using a pre-emergence herbicide (Pendimetalin 322 g L^−1^ + Imazetapir 22 g L^−1^) at a rate of 2 L ha^−1^. Neither irrigation nor pesticide usage were included in the experimental scheme. The chemical and physical characteristics of soil were: 33% clay, 19% silt and 48% sand, pH 6.8, 0.96% organic matter, and 0.054% total N. In both years chickpea was grown after durum wheat in a two-year rotation system.

**Figure 1:**
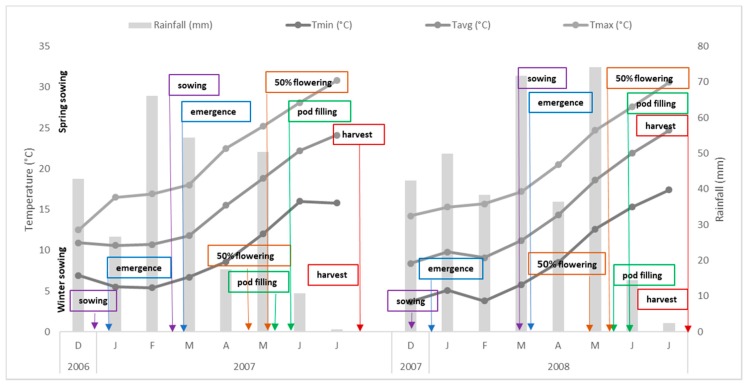
Weather pattern and phenological stages of chickpea during the two growing seasons. Downward labels are referred to winter sowing (E1); upward labels are referred to spring sowing (E2).

For both Y1 and Y2, the mean air temperature during the growing seasons was, 16 ± 5.5 °C for winter sowing and about 18 ± 4.7 °C for spring sowing. Looking at winter sowing, the total rainfall registered during the chickpea growing season was 227 mm, and 307 mm for Y1, and Y2, respectively; whereas, about spring sowing, it was 134 mm, and 162 mm for Y1, and Y2, respectively. Weather patterns with chickpea phenological phases are shown in Figure 1.

### 2.2. Harvesting and Sample Preparation 

Harvesting was carried out using a plot harvester on 30 July 2007 for Y1, and 1st August 2008 for Y2, after physiological maturity, when about 90% of plants were completely dry. After thorough cleaning and removal of undesired material, the grains were stored in paper envelopes at room temperature (22 ± 2 °C) for 7 days and then milled.

### 2.3. Analytical Procedures

Seeds were dried at 65 °C for 48 h in a forced air oven and then grinded through a mill (Retsch, Haan, Germany) to pass 1 mm screen. After thorough mixing, dry milled samples were stored in sealed polyethylene bottles until analysis. 

Sucrose, maltose, raffinose family oligosaccharides (raffinose, stachyose) and galactosyl cyclitols (galactinol and ciceritol) were determined according to the RP-HPLC procedure described by Muzquiz et al. [26]. The standard solutions for external calibration were obtained from Sigma-Aldrich Co. (St. Louis, MO, USA). Ciceritol was purified and kindly supplied by Dr. A. I. Piotrowicz-Cieslak (Olsztyn-Kortowo, Poland). The results were expressed as g kg^−1^ on dry matter (DM) basis. All the samples were analyzed in duplicate.

Trypsin inhibitors (TI) were determined following the procedure described by Kakade et al. [27], using α-n-benzoyl-DL-arginine-p-nitroanilide hydrochloride (Sigma-Aldrich Co., St. Louis, MO, USA) as substrate. The results were expressed as Trypsin Inhibitor Units (TIU) per mg of protein [27]. Spectrophotometric determinations were carried out at 410 nm by a UV-1601 double beam spectrophotometer (Shimadzu Corp., Kyoto, Japan) against negative control.

Inositol trisphosphate (IP3), inositol tetraphosphate (IP4), inositol pentaphosphate (IP5), and inositol hexaphosphate (IP6 or phytic acid) were extracted and determined, as described by Cuadrado et al. [28] with minor modifications. The total inositol phosphates content (TIPs) was the sum of the compounds listed above. In brief, chickpea flour (500 mg) was extracted with 10 mL of 0.5 M hydrogen chloride (Sigma-Aldrich Co., St. Louis, MO, USA) for 1 min using an Ultra-Turrax^®^ (T25 basic, IKA, Königswinter, Germany) homogeniser operated at 9,000 rpm, then centrifuged for 15 min at 27,000 rpm (4 °C). After that, 25 mL of distilled water were added to 5 mL of supernatant and the diluted extract was eluted through 3 mL SAX columns (Varian, Harbor City, CA, USA). The Ips were recovered with 2 mL of 2 M HCl, vacuum dried and then suspended in 0.5 mL of buffer solution (51.5% methanol, 48.5% milliQ-water, 1.6 mL TBNOH—40% in milliQ-water, 0.2 mL H_2_SO_4_ 5 M and 0.1 mL formic acid 91%). The samples were injected in an ion-pair HPLC system (Beckman System Gold Instrument, Los Angeles, CA, USA) equipped with a macroporous (150 × 4.1 mm i.d., 5 µm) polymer PRP-1 column (Hamilton, Reno, NV, USA), maintained at 45 °C and with a flow rate of 1 mL min^−1^. The mobile phase was 0.012 M formic acid in 51.5% methanol and 0.8% TBA-OH, adjusted to pH 4.3 with sulfuric acid. The results were expressed as gram of standard phytic acid (Sigma-Aldrich Co., St. Louis, MO, USA) per kg DM.

### 2.4. Statistical Design and Analysis

The response variables measured were subjected to ANOVA, using a year-combined complete block design (Trial 1); a separate ANOVA was performed, comparing data collected in Trial 2 to verify the effect of seeding rate. The differences between means were compared by Fisher’s least significant difference (LSD) test. Significance was always declared at *p* ≤ 0.05, unless noted otherwise. Data analyses were performed using R 3.5.3 software [29]. 

## 3. Results

### 3.1. Trial 1

The content of bioactive compounds in chickpea seeds is reported in Table 1. 

Significant effects of cultivar, growing season, time of sowing and their interaction were detected for some of the traits studied. The two cultivars showed marked differences of some α-galactosides, but not in the sum of them. Sucrose was more represented (*p* < 0.01) in Pascià (19.4 g kg^−1^), compared to Sultano (15.1 g kg^−1^), while ciceritol and raffinose were greater (*p* < 0.01) in Sultano (35.5 kg^−1^, and 5.6 g kg^−1^, respectively) than in Pascià (29.4 kg^−1^ and 5.0 g kg^−1^, respectively). Neither IPs nor trypsin inhibitors showed significant differences between cultivars. The growing season also affected some α-galactosides, such as raffinose, ciceritol, and TSGs, which reached a greater (*p* < 0.05) value in Y1 (81.7 g kg^−1^) with respect to the Y2 growing season (74.8 g kg^−1^). IP5 was higher in Y1 (1.2 g kg^−1^) than in Y2 (0.9 g kg^−1^) (*p* < 0.01), but for the IP6 a lower value (*p* < 0.01) was recorded in Y1 (7.5 g kg^−1^) with respect to Y2 (8.0 g kg^−1^). Overall, the content of inositol phosphates did not differ significantly between the two trials. The accumulation of trypsin inhibitors was higher in Y1 than in Y2 (20.2 versus 13.4 TIU mg protein^−1^). As for the effect of sowing time, the amount of maltose and ciceritol was greater in the winter sowing (E1, 3.0 and 32.8 g kg^−1^, *p* < 0.01, *p* < 0.05, respectively) compared with spring sowing (E2, 2.3 and 32.1 g kg^−1^). The same trend was observed for IP6 (7.4 and 6.6 g kg^−1^), TIPs (8.8 and 7.9 g kg^−1^) and TI (18.6 and 15.1 TIU mg protein^−1^). Significant effects due to cultivar × growing season, cultivar × time of sowing, and cultivar × year of sowing x time of sowing interactions were also observed, suggesting a certain variability for the studied parameters between the two genotypes and a relative phenotypic response over environmental condition.

**Figure 2:**
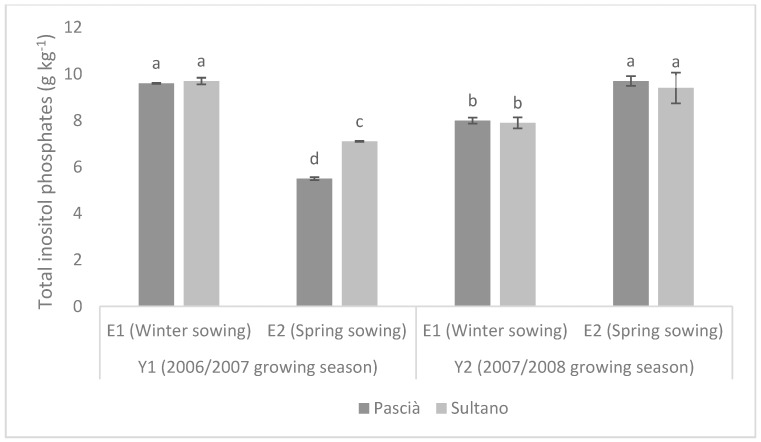
Content of Total inositol phosphates in seeds (g kg^−1^ ± SE) of the two varieties in the two growing seasons. Means with different letters are significantly different (Fisher’s LSD, *p* ≤ 0.05).

TIPs content showed an opposite trend between 2006–2007 and 2007–2008 growing seasons (Figure 2). In seeds harvested in 2007 (Y1), TIPs were significantly higher for winter sowing than for spring sowing, with Sultano having higher TIPs level than Pascià if sown in spring. Conversely, TIPs were significantly higher for spring planting than winter one in 2008, with similar level between Sultano and Pascià for both planting seasons.

**Figure 3:**
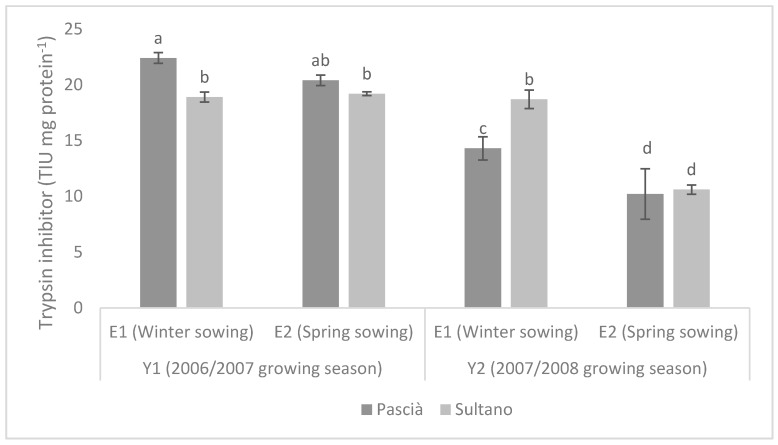
Content of Trypsin inhibitors in seeds (TIU mg protein ± SE) of the two varieties in the two growing seasons. Means with different letters are significantly different (Fisher’s LSD, *p* ≤ 0.05).

Planting season did not affect TI level for Pascià and Sultano in Y1 while it did it in Y2, when winter sowing resulted in significantly higher TI values than spring one (Figure 3). 

### 3.2. Trial 2

Bioactive compounds content is reported in Table 2.

Many of the results, obtained in Trial 1, were confirmed, highlighting substantial differences between the two varieties, and between sowing seasons. 

Sultano was confirmed to be a major accumulator of ciceritol (30.4 g kg^−1^), Pascià (26.8 g kg^1^), and it had also greater (*p* < 0.01) level of TI (15.2 vs. 13.3 TIU mg protein^−1^). As in Trial 1, when compared with spring sowing, winter sowing enhanced (*p* < 0.01) the content of maltose (3.1 versus 2.4 g kg^−1^), galactinol (2.6 versus 2.1 g kg^−1^), ciceritol (29.5 versus 27.7 g kg^−1^), and TI (16.1 versus 12.3 TIU mg protein^−1^), but it reduced (*p* < 0.01) the raffinose content (5.0 versus 5.5 g kg^−1^). In contrast with what observed in Trial 1, winter sowing significantly reduced (*p* < 0.01) the TIPs content (8.0 g kg^−1^) of chickpea grains with respect to spring sowing (9.3 g kg^−1^), and the synthesis and accumulation of IP5 and IP6 was mainly affected by sowing time. The lowest seeding rate (70 seeds m^−1^) resulted in a major (*p* < 0.01) accumulation of sucrose, raffinose, stachyose, and TSGs (16.4, 5.8, 18.3, and 74.8 g kg^−1^, respectively), compared with the high seeding rate (13.6, 4.7, 16.5, and 67.9 g kg^−1^, respectively), without appreciable effects on the other traits.

The interactions between cultivar, sowing time, and seeding rate were not significant, and only TI and stachyose seemed to be affected by the interaction between sowing date × seeding rate, and among cultivar × sowing, date × seeding rate, respectively.

## 4. Discussion

It has been shown that leguminous plants, including chickpea, accumulate raffinose family oligosaccharides in seeds, during yellowing and desiccation [30], and after physiological maturity [31]. These metabolites, that act as a reserve carbohydrate, play a central role in carbon allocation and modulate some physiological processes (i.e., cold and desiccation tolerance) [32]. The RFOs are synthesized de novo in legumes, and accumulate progressively from flowering until drying [33,34]. However, their concentration in legume seeds can depend on the cultivar, as well as the growing conditions (i.e., soil type, fertilizer application, water shortage, air temperatures, etc.) [35].

Regarding the cultivar, we noticed that Pascià contains more sucrose and less ciceritol than Sultano, but this is of minor importance, as they are not flatulence factors. In quantitative terms, ciceritol and stachyose are the most represented, but only the stachyose concentration in chickpea grains was found to be plant density-dependent, as demonstrated in Trial 2. Since the ciceritol does not cause flatulence in monogastrics, because its chemical structure (galactosyl cyclitols) promotes hydrolysis [36], only stachyose can exert an anti-nutritional effect. However, Sandberg et al. [37] demonstrated that stachyose, as well as raffinose, are partially degraded and digested in the stomach and small intestine of humans, and because they pass mostly undigested into the lower gut, they behave as dietary fibres acting as a prebiotic, also in monogastric animals [38]. 

Findings obtained with Trial 2 showed that a sparser seeding rate lead to a higher concentration of RFOs (raffinose and stachyose, in particular). This can be partially explained by the fact that a denser canopy tends to decrease the photosynthetic capacity, because many leaves do not experience saturating light intensities, especially when canopies present a full closure. This, in addition to the competition for nutrients and water uptake, which are most noticeable at high seeding rate, can affect the biosynthesis of the precursor sucrose during the early development of seeds [31] and, later, during maturation and desiccation, the RFOs synthesis, and accumulation.

In terms of the trypsin inhibitors, the data presented in this study are consistent with other findings that indicate that winter varieties of pulses [39] and wheat [40] can have more defensins (including protease inhibitors) in response to cold induction and acclimation than spring varieties. Seeds accumulate protease inhibitory compounds as they reach maturity, since they act as defensins [41], protecting the plant and, in particular, the seeds from predation, and against fungi and other microorganisms, also during storage [42]. The fact that legume grains accumulate protease inhibitory compounds late in the season could explain the lower content of trypsin inhibitors, detected in chickpea grains, harvested in 2008 than 2007. In fact, the high biotic stress caused by *Ascochyta blight* (AB) from April to early June 2008 [23], besides determining a significant yield loss (−54%), could have also caused a low efficiency in the accumulation of such compounds. Consequently, the decrease in TI content was more marked in the susceptible cultivar Pascià, than in the more resistant Sultano. It has to be noted that, in 2008, the disease pressure was substantially greater than in 2007, due to almost 50% more rainfall during reproductive development (April and May). Specifically, on May 20th and 21st (2008) 80 mm-rainfall occurred and maximum relative air humidity (RH) was constantly higher than 95% from May 18th to May 24th. In that period, plants were in the middle of flowering phase (50% flowering detected on May 10th) which, together with podding, is the most susceptible growing stage [43,44]. In field experiments conducted in India, the dynamics of AB infection clearly evidenced a more rapid disease progress in plants inoculated at flowering than other stages [44]. Additionally, during the reproductive phase, the temperature range was 8.6–31.8 °C with a mean of 18 °C, and average RH varied from 44.8 to 92.3%, so the environmental conditions would often have been within the optimum range for AB, as previously indicated by Jhorar et al. [45]).

A combined effect of cultivar, growing season and sowing time on TIPs and TI content has been found in this study, suggesting that these compounds were markedly dependent on the growing environmental conditions, and the interaction between genotype and the environment must be accurately considered. Even though we did not find any previous research investigating simultaneously the role of growing year, cultivar, and planting season on chickpea anti-nutritional factors, the effects, involving year and planting season, could be related to rainfall and seasonal pattern of air temperature, which are the most obvious environmental data changes between the two growing seasons encompassed by this study. For instance, rainfall has been positively related to trypsin inhibitors in cowpeas farmed in Nigeria [46]. Consistently with those results, we found a significantly higher TI content in winter planting (256.9 ± 30.3 mm rainfall), as compared to spring (166.4 ± 32.6 mm rainfall). However, in contrast with findings from Oluwatosin [46], we found a significantly lower TI content in 2007–2008 growing season when total rainfall was higher than in 2006–2007 growing season (287.2 mm, and 226.6 mm, respectively). These contrasting results could be attributable to the difference in total rainfall amount, which was about 100 mm between winter and spring sowing in both years, while it was from 30 mm to 50 mm between 2006–2007 and 2007–2008 growing seasons (winter, and spring sowing, respectively). 

In terms of TIPs content, environmental conditions markedly affected this trait, but results were inconsistent between years. Clearly there are major shortcomings in the ability to adequately predict the accumulation of anti-nutrient compounds in chickpea seeds on the basis of climatic conditions. Nikolopoulou et al. [25] also found contrasting results in phytic acid and total tannins content for three chickpea cultivars, in two subsequent seasons in Greece. Additionally, a complex and contrasting relationship was found between anti-nutritional factors and soil characteristics for the genus *Vicia* in Western Australia [47]. These findings suggest that, to better understand how climatic conditions influence anti-nutritional content in grain legumes, multi-year and location trials should be conducted. However, the effect of year, planting season, and variety indicate that the regulation of anti-nutrients in grain legumes might be extremely difficult. In addition, plant defense responses to pathogens (i.e., to *A. rabiei*), through the synthesis of bioactive compounds with anti-nutritional effects involve a very specific metabolic event, multigenic governed by resistance-quantitative trait loci (R-QTL), where some metabolic pathways are still not well known [48].

## 5. Conclusions

Even though just two varieties of Kabuli chickpeas were tested, this study has shown that the concentration of some bioactive compounds is influenced by genotype, climatic conditions, agronomic techniques and their interaction, leading to considerable variability in the qualitative characteristics of this traditional pulse. Spring sowing appeared to be the best choice when cultivating to minimize some α-galactosides and trypsin inhibitors in the Mediterranean environment. However, winter sowing determines an overall increase in seed yield (+19%) and total protein amount per hectare as already demonstrated by us. This demonstrates that a complex trade-off between quality and quantity of chickpea grain has to be faced when choosing agronomic strategies, such as planting date and cultivar selection. Seeding rate had a relatively little effect but data from multi-year trials are needed to draw solid conclusions on this aspect.

Further studies will be focused on the effect of anti-nutritional factors occurring in the chickpea lines, studied on animal nutrition and performance, by in vitro and in vivo trials, and to assess the relationship between their biosynthesis in plants and infection from *A. rabiei*. This will provide further evidence to assist in selecting the best chickpea cultivar, cultivation practices, and animal feeding approaches.

## Figures and Tables

**Table 1:** Trial 1: Effects of cultivar (Pascià versus Sultano), growing season (Y1 versus Y2), and sowing date (E1 versus E2) on the content of galactosides and inositol phosphates (g kg^−1^ DM), and trypsin inhibitors (TIU mg protein^−1^) in chickpea grains.

	Cultivar (C)			Growing Season (Y)			Sowing Date (E)			Interactions (*p*-Level)			
	P	S	*p*	Y1	Y2	*p*	E1	E2	*p*	C × Y	C × E	Y × E	C × Y × E
Sucrose	19.4	15.1	**	18.2	16.4	ns	17.5	17.0	ns	**	ns	ns	ns.
Raffinose	5.0	5.6	**	4.8	5.8	**	5.2	5.4	ns	ns	**	**	ns
Stachyose	18.9	17.2	ns	17.8	18.4	ns	17.7	18.4	ns	ns	ns	ns	ns
Maltose	2.5	2.8	ns	2.5	2.7	ns	3.0	2.3	**	**	ns	ns	*
Galactinol	2.5	2.5	ns	2.6	2.4	ns	2.7	2.3	ns	*	ns	ns	ns
Ciceritol	29.4	35.5	**	35.7	29.1	**	32.8	32.1	*	**	**	*	ns
TSGs	77.7	78.8	ns	81.7	74.8	**	79.0	77.5	ns	ns	*	ns	ns
IP3	0.1	0.1	ns	0.1	0.1	ns	0.1	0.1	ns	ns	ns	ns	ns
IP4	0.3	0.2	ns	0.3	0.2	ns	0.2	0.2	ns	ns	ns	ns	ns
IP5	1.0	1.0	ns	1.2	0.9	**	1.1	1.0	ns	ns	ns	**	ns
IP6	6.8	7.1	ns	6.4	7.5	**	7.4	6.6	**	*	ns	**	*
TIPs	8.2	8.5	ns	8.0	8.8	ns	8.8	7.9	**	*	ns	**	*
TI	16.8	16.9	ns	20.2	13.4	**	18.6	15.1	**	ns	ns	*	*

P = Pascià, S = Sultano; Y1 = 2006–2007, Y2 = 2007–2008; E1 = winter, E2 = spring; TSGs = total sugars and galactosides; IP3 = inositol trisphosphate; IP4 = inositol tetraphosphate; IP5 = inositol pentaphosphate; IP6 = inositol hexaphosphate; TIPs = total inositol phosphates; TI = trypsin inhibitors. * *p* < 0.05; ** *p* < 0.01; ns = not significant.

**Table 2:** Trial 2: Effects of cultivar (Pascià versus Sultano), sowing date (E1 versus E2) and sowing rate (low versus high) on the content of galactosides and inositol phosphates (g kg^−1^ DM) and trypsin inhibitors (TIU mg protein^−1^) in chickpea grains.

	Cultivar (C)		*p*	Sowing Date (S)		*p*	Seeding Rate (R)		*p*	Interactions (*p*-Level)			
	P	S		E1	E2		L	H		C × S	C × R	S × R	C × S × R
Sucrose	16.2	13.8	ns	14.9	15.1	ns	16.4	13.6	**	ns	ns	ns	ns
Raffinose	5.0	5.4	ns	5.0	5.5	**	5.8	4.7	**	ns	ns	ns	ns
Stachyose	18.2	16.5	ns	16.9	17.8	ns	18.3	16.5	**	ns	ns	ns	**
Maltose	3.2	2.3	ns	3.1	2.4	**	2.7	2.7	ns	ns	ns	ns	ns
Galactinol	2.7	2.1	ns	2.6	2.1	**	2.4	2.4	ns	ns	ns	ns	ns
Ciceritol	26.8	30.4	**	29.5	27.7	**	29.1	28.0	ns	ns	ns	ns	ns
TSGs	72.2	70.4	ns	72.0	70.7	ns	74.8	67.9	**	ns	ns	ns	ns
IP3	0.1	0.1	ns	0.1	0.1	ns	0.1	0.1	ns	ns	ns	ns	ns
IP4	0.2	0.2	ns	0.2	0.2	ns	0.2	0.2	ns	ns	ns	ns	ns
IP5	1.0	0.9	ns	0.8	1.0	**	0.9	0.9	ns	ns	ns	ns	ns
IP6	7.6	7.3	ns	6.8	8.0	**	6.8	8.0	ns	ns	ns	ns	ns
TIPs	8.9	8.5	ns	8.0	9.3	**	8.8	8.6	ns	ns	ns	ns	ns
TI	13.3	15.2	**	16.1	12.3	**	13.4	15.0	ns	ns	ns	**	ns

P = Pascià, S = Sultano; E1 = winter, E2 = spring; L = 70 seed m^−2^, H = 110 seeds m^−2^; TGs = total sugars and galactosides; IP3 = inositol trisphosphate; IP4 = inositol tetrakisphosphate; IP5 = inositol pentakisphosphate; IP6 = inositol hexaphosphate; TIPs = total inositol phosphates; TI = trypsin inhibitors. * *p* < 0.05; ** *p* < 0.01; ns = not significant.
